# Patterns of livestock activity on heterogeneous subalpine pastures reveal distinct responses to spatial autocorrelation, environment and management

**DOI:** 10.1186/s40462-015-0053-6

**Published:** 2015-10-08

**Authors:** Hermel Homburger, Andreas Lüscher, Michael Scherer-Lorenzen, Manuel K. Schneider

**Affiliations:** Agroscope, Institute of Sustainability Sciences, Reckenholzstrasse 191, CH-8046 Zurich, Switzerland; University of Freiburg, Faculty of Biology, Geobotany, Schaenzlestrasse 1, D-79104 Freiburg, Germany

**Keywords:** GPS tracking, Grazing, INLA, Spatial autocorrelation, Stocking rate, Alps, Animal behavior, Pasture ecology

## Abstract

**Background:**

In order to understand the impact of grazing livestock on pasture ecosystems, it is essential to quantify pasture use intensity at a fine spatial scale and the factors influencing its distribution. The observation and analysis of animal activity is greatly facilitated by remote tracking technology and new statistical frameworks allowing for rapid inference on spatially correlated data. We used these advances to study activity patterns of GPS-tracked cows in six summer-grazing areas in the Swiss Alps that differed in environmental conditions as well as livestock management.

**Results:**

Recorded GPS positions were assigned to the activities of grazing, resting, and walking, and were discretized on a regular grid. Regression models with spatially structured effects were fitted to the spatial activity patterns using Integrated Nested Laplace Approximation. They indicated that terrain slope, forage quality, and stocking rate were the primary factors determining cow activity in the six study areas. Terrain slope significantly reduced livestock activity in five of the six areas and sparse forage availability significantly reduced grazing in all areas. In three areas, grazing pressure imposed by the pasture rotation was observable in the grazing pattern. Insolation, distance to the shed, and distance to water were less important for cow activity. In addition to the main factors identified across all study areas, we found effects operating only in individual areas, which were partly explained by specific environmental and management characteristics. In study areas with few paddocks, environmental variables exerted a stronger control on livestock activity than in areas with a short stocking period per paddock.

**Conclusions:**

The data demonstrated that a strict pasture rotation with short stocking periods is necessary to influence livestock activity, and hence potential effects on ecosystem processes. Without grazing management, livestock activity is primarily determined by the environment. Such insight is indispensable for studying relationships between grazing animals and ecosystem characteristics, and for developing management strategies to optimize ecosystem services. The analysis also highlighted the need for an appropriate statistical treatment of bio-logging data, since various estimates were biased if spatial autocorrelation was ignored.

**Electronic supplementary material:**

The online version of this article (doi:10.1186/s40462-015-0053-6) contains supplementary material, which is available to authorized users.

## Background

A quarter of the global land surface is covered by managed grasslands and many of them are strongly influenced and structured by grazing livestock [1]. The intensity of pasture use is a primary driver of grassland ecology and related ecosystem services [2–4], and consists of a set of distinct activities of the animals. For example, forage intake by grazing determines vegetation structure and composition [5–7], and trampling affects various soil properties, especially soil stability [8]. Additionally, resting places of livestock are typically intensively trampled down and defecated on, which has large impacts on vegetation and soil nutrient content [9].

Patterns of livestock activity arise from environmental variation and from livestock management by humans. In topographically heterogeneous landscapes, livestock tend to replicate long-existing activity patterns, e.g. by increased grazing and defecation on patches with palatable forage [10]. Farmers aim at counterbalancing the influences of environmental drivers on pasture use, to a certain degree, by means of herding the animals, fencing, strategic placement of water or nutrient supplements, or even by the construction of sheds and access roads [11]. Hence, many environmental constraints are modifiable by human intervention, albeit requiring various levels of effort. The result is a continuum of pasture properties from those that are hardly modifiable (e.g. terrain slope) to those that are more easily managed (e.g. paddock rotation by fencing).

Livestock management by farmers has changed over time due to structural developments in mountain agriculture, which vary regionally [12]. For example, the number of people employed in agriculture in the Swiss Alps has decreased by one half in the last 30 years (Swiss Statistics, Neuchâtel, Switzerland), resulting in less available labor for livestock management and pasture maintenance [13]. In present practice in marginal grasslands, which require high labor input, farmers often exercise limited control over the animals’ distribution. An example of such grasslands are summer pastures in the European Alps, which have been grazed by domestic animals for centuries and where livestock activity patterns and associated ecosystem characteristics are prone to respond to structural changes in mountain agriculture [14, 15].

In consideration of the agricultural development, analyzing spatial patterns of animal movement in heterogeneous terrain is crucial to understanding drivers of animal activity and consequences for the ecosystem. However, quantifying the relationship between livestock activity patterns and site conditions presents several challenges. Specifically, animal activity should be measured directly, and the data should be gathered at an appropriate scale that supports the aim of the research. There are two common approaches to quantifying animal activity: it can be deduced from ecosystem properties, such as vegetation composition, or by counting bitten plant shoots [16]. These indirect methods have the inherent problem that the measured animal activity is not independent from eventual explanatory variables. A frequently used alternative is the direct quantification of pasture use intensity, such as the average stocking rate of animals per paddock [17]. However, such data are frequently at a hectare-scale and neglect changes in intensity over short distances. Gathering data at a finer spatial scale, however, is especially important in mountainous regions, where various site conditions, such as terrain slope, differ at a scale of meters. Because visual observation of animals is highly time consuming and observer-dependent, and animal behavior can be influenced by the physical presence of an observer, bio-logging techniques, such as GPS tracking, offer great advantages [18]. The resulting position data offer the possibility to determine livestock behavior while at pasture [19], as well as to quantify spatial activity patterns.

In certain applied studies that sought to shift grazing activity to less favored areas [20, 21] or to promote opening of shrub-encroached pastures [11, 22], the distribution of grazers within heterogeneous landscapes was investigated, often based on the placement of mineral supplement or water supply. In these and related studies, livestock distributions were recorded by counting the number of animal visits at certain locations or in landscape units, and were analyzed by calculating preference indices [22–25] or home ranges of the grazing animals [11, 21]. Several recent studies dealing with wild ungulates used complex regression models of animal distribution with one or more empirical covariates and accounted for spatial autocorrelation of the data [26–29]. However, almost all of them considered only one, albeit sometimes large, study area and did not specifically address differences between areas in, for example, environmental setting and management strategies.

Our aim, therefore, was to determine driving factors of pasture use intensity by cows in heterogeneous areas with distinct environmental conditions and livestock management. Specifically, three sets of questions and hypotheses were addressed:

1. How strong is the influence of different environmental and management covariates on fine-scale spatial patterns of the three predominant cow behaviors of grazing, resting, and walking? How do estimated covariate effects differ with regard to the entire grazing period, individual animals, time of the day and season? Our hypothesis was that, since the primary objective of grazing is forage intake, this activity would be strongly affected by availability and palatability of vegetation. Since lactating dairy cows consume considerable amounts of drinking water, the distance to water sources was also assumed to affect the spatial distribution of grazing. In contrast, resting and walking were expected to be influenced by topographic properties such as terrain slope, insolation, or distances to the shed. We expected that individual variation, daytime and season may affect the response to environmental conditions to some degree, but that main effects are generally maintained.
2. Which drivers of animal activities are common to all study areas and which only act in particular settings? Can specific characteristics of the study areas explain varying effects between areas? We hypothesized that human management actions would modify effects of environmental drivers on livestock activity, e.g. by fencing or the strategic placement of water sources. Stronger human intervention, e.g. a rapid rotation in many paddocks, should result in more even activity patterns and, hence, diminish the influence of environmental constraints.
3. How sensitive are the estimated covariate effects to spatial autocorrelation and its specification? We hypothesized that considering spatial autocorrelation would be important in the analysis of activity patterns and that estimated effects of spatially strongly correlated covariates would be most sensitive to model specification.

**Fig. 1:**
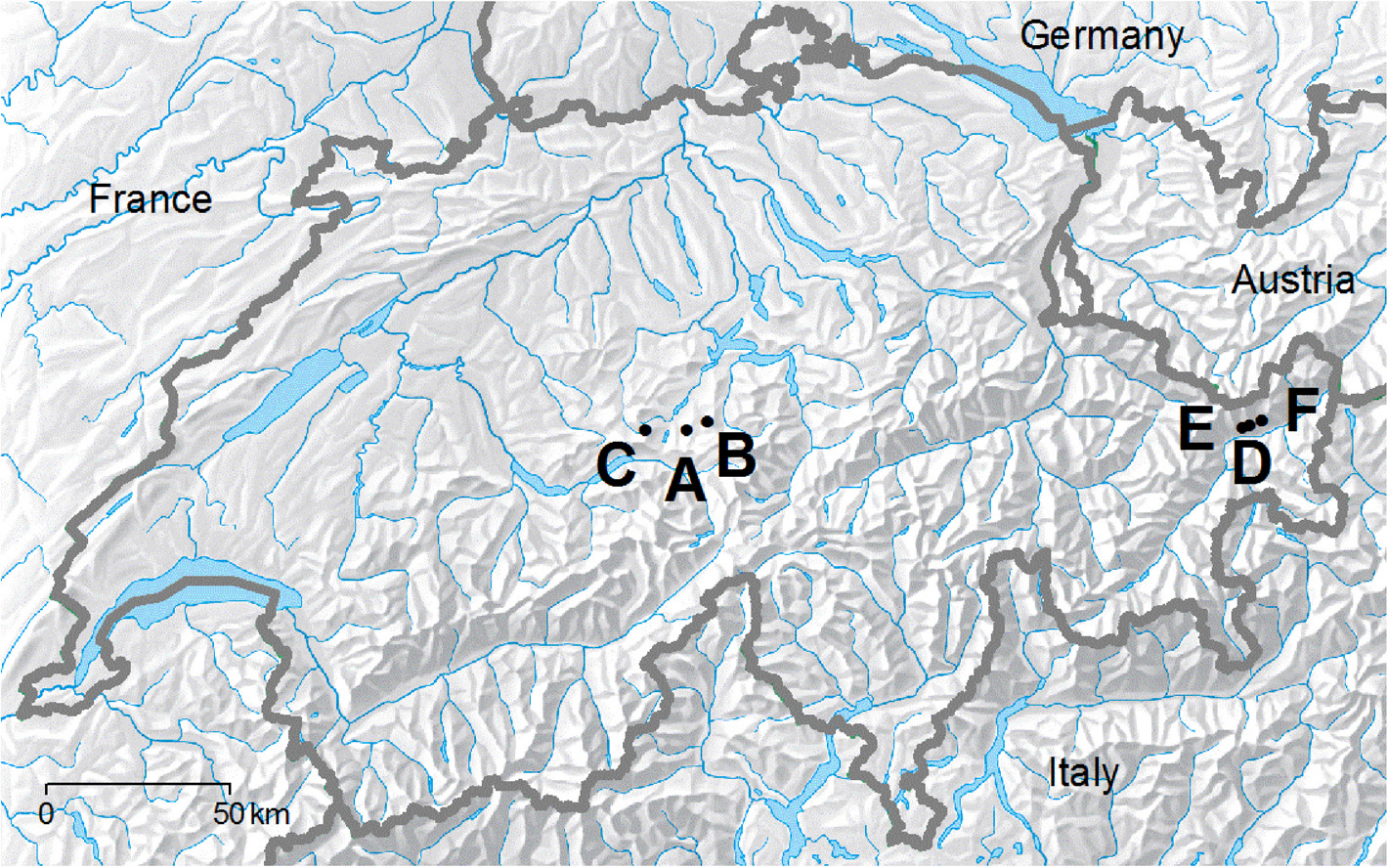
Location of the study areas in the Swiss Alps. Areas A-C are located in the Obwalden region, areas D-F in the Lower Engadine. The background map is copyright of swisstopo, Wabern, Switzerland

To test these hypotheses, we tracked positions of selected cows at a temporal resolution of 20 s in six study areas in two regions of the Swiss Alps for an entire grazing season (Fig. 1) and analyzed activity patterns by spatial regression using Integrated Nested Laplace Approximation (INLA) [30]. In order to discern common and area-specific drivers of livestock activity, the estimated covariate effects were related to characteristics of the study areas.

## Methods

### Study areas

The study was conducted on six temporally-grazed upland areas, so-called alpine farms, A to F, at elevations between 1,300 and 2,300 m asl (Table 1). Study areas D-F were at higher elevations due to inner-alpine climate conditions. All six study areas were grazed by dairy cows and areas B and D also had suckler cows. The study areas were situated in two Swiss mountain regions: areas A-C in the canton of Obwalden in the Northern foothills of the Alps, areas D-F in the Lower Engadine (canton of Grisons) in the Eastern Central Alps (Fig. 1). Geology of the study areas was dominated by calcareous bedrock in Obwalden and by silicates in the Lower Engadine. The vegetation was mainly composed of montane and subalpine grassland types, dwarf shrub communities, and few small pasture areas with open forest. In Obwalden, dairy cows spent the night in the shed, whereas in the Lower Engadine they were held in small paddocks near the farm building during night. Salt was provided to the animals in the shed during milking. The size of single paddocks varied between 0.17 ha and 87 ha, and herd size varied between 30 and 120 cows (Table 1). Herd sizes and paddocks were generally smaller in the study areas A-C than in the study areas D-F. In each of the two regions, the three areas differed in the degree to which livestock activity was controlled by fencing and paddock rotation, so that the average stocking period per paddock ranged from 3.3 to 14 days in the Obwalden region and from 8 to 11 days in the Lower Engadine region.

**Table 1:** Characteristics of the six study areas

Area	A	B	C	D	E	F
Region	Obwalden	Obwalden	Obwalden	Lower Engadine	Lower Engadine	Lower Engadine
Longitude (°)	8.241	8.31	8.097	10.182	10.157	10.223
Latitude (°)	46.813	46.832	46.813	46.794	46.79	46.805
Elevation (m asl)	1862 ± 127	1440 ± 142	1585 ± 73	2206 ± 109	2163 ± 160	2101 ± 184
Slope (%)	53 ± 20	40 ± 15	38 ± 10	42 ± 11	41 ± 12	36 ± 13
Mean annual temperature (°C)	2.7	4.8	3.9	0.2	0.3	1.4
Mean annual precipitation (mm)	1949	1822	1801	1206	1206	1285
Size (ha)	56	40	25	91	135	158
Herd size (LU)	30	45	42	55	50	120
Simultaneously tracked animals	3	2	2	3	3	4
Number of records	245752	122746	157962	438677	485620	483175
Grazing period (d)	69	90	90	43	72	78
Number of paddocks	5	7	27	4	7	10
Average stocking period/paddock (d)	13.8	12.9	3.3	10.8	10.3	7.8
Stocking rate (LU ha^−1^ yr^−1^)	0.10 ± 0.06	0.16 ± 0.16	0.25 ± 0.23	0.04 ± 0.05	0.05 ± 0.06	0.09 ± 0.06

Values for elevation, slope and stocking rate are medians ± standard deviation. Mean annual precipitation was derived from grid data of MeteoSwiss, Zurich. Mean annual temperature was taken from Hiebl et al. [48]

### Measuring grazing, resting, and walking intensity by GPS tracking

In 2011, we equipped three to four cows in each area with GPS collars. To make sure that these animals represented the entire herd as best as possible, we selected animals that were well integrated into the herd, according to the farmers. Only dairy cows were tracked, with the exception of the suckler cows in area B, where suckler cows alone grazed a large part of the pasture area that was included in the study. In area D, suckler cows were not tracked, because they grazed outside of the studied pasture area and were only let out to graze the entire farm area at the end of the grazing season. The breed of most of the tracked dairy cows was Swiss Braunvieh, except for one Jersey and one Red Holstein cow. The suckler cows were Angus cattle. For technical reasons, tracked cows were occasionally changed during the grazing season. A small leather saddle carrying the logger (Qstarz BT-Q1000XT, Qstarz Ltd., Taipei, Taiwan) was mounted directly on the bell collar of each cow selected for study. GPS positions were collected from June to September 2011, with a recording interval of 20 s.

In order to classify the dataset into different activities, the behavior of each cow equipped with a GPS collar was observed during several hours in the field and the protocolled activities were assigned to the corresponding positions. A random forest algorithm was trained to discern the activities of grazing, resting, and walking based on 102 movement metrics calculated across multiple GPS positions as predictor variables [19]. The evaluation showed that movement speed from one position to the next was important for discriminating walking from other activities. However, speed values averaged across time steps larger than 20 s were needed to separate resting and grazing, the latter often being associated to a slow but continuous movement with short stops. Because the activities of grazing, resting, and walking were unequally frequent, training data was balanced by random under-sampling. By this method, the occurrence of all three activities in independent validation sequences was predicted with an average accuracy of 77 % (grazing: 82 %, walking: 68 %, resting: 68 %). Without balancing, classification was more accurate overall but strongly activity-specific (average: 83 %, grazing: 95 %, walking: 36 %, resting: 58 %). With our data, the random forest algorithm achieved a higher classification accuracy than alternative techniques such as linear discriminant analysis, support vector machines and state-space models [19].

The classified GPS positions were discretized on a grid of 25 m × 25 m cells, aligned to the digital terrain model (DHM25, Federal Office of Topography swisstopo, Wabern, Switzerland). This grid resolution was selected based on practical and ecological considerations. For example, the absolute measurement accuracy of the GPS devices is approximately 3 m [19]. In addition, vegetation was mapped in polygons larger than 400 m^2^. Furthermore, we were interested in the distribution patterns of cow activities over a whole grazing season and at a scale of about 10 to 30 m, rather than in micro-scale processes, such as the selection of individual plants.

In all six study areas, cows spent time inside the shed for milking. In regions A-C, dairy cows also spent the night in the shed. Positions recorded during these periods were inaccurate and therefore discarded. Due to the occasional harsh climatic conditions in the study areas, temporary failures of some measuring devices occurred. In area B, GPS data in summer 2011 were insufficient for statistical analysis. However, we achieved better data for area B by repeating GPS tracking in 2012, and because the cows change from paddock to paddock in the same order each year, we combined the datasets from both years for this study area.

### Calculation of environmental and management covariates

The effects of seven possible covariates influencing grazing, resting, and walking intensity were evaluated: elevation, terrain slope, insolation, forage quality, distance to the shed, distance to nearest water source, and stocking rate. The seven covariates represented different degrees of intervention by the farmers: in most cases there is essentially no human control of elevation, terrain slope, and insolation, little control of vegetation and the placement of the shed or the milking parlor, and a high level of influence on the placement of water sources and on pasture rotation. Elevation and terrain slope were extracted from elevation data with a resolution of 25 m (DHM25, swisstopo, Wabern, Switzerland), potential incoming solar radiation (insolation) was calculated for each grid cell based on slope, aspect, and location using SAGA 2.1.1 (University of Hamburg, Germany). Vegetation was mapped over the whole surface of each area during summers 2011 and 2012 at the level of the phytosociological alliance, according to the code of Delarze [31]. The smallest patch size considered for mapping was 400 m^2^. Based on analyses of plant nutritive value in Swiss alpine pastures [32], the 22 vegetation types mapped (Additional file 1) were reclassified into three categories of forage quality, specifically (i) nutrient-poor vegetation, (ii) nutrient-rich vegetation, and (iii) vegetation with sparse forage supply to livestock (forest, shrub, rock, sedge). In area B, the nutrient-poor vegetation category was too rare for inference and was merged with nutrient-rich vegetation. Distance to shed was calculated for each grid cell from its center point. Locations of water ponds or other water sources were mapped in each study area and the distance to the nearest water source was also calculated from the center point of each grid cell. Farmers recorded the number of animals and the time they spent in each paddock during the grazing season, from which the average stocking rate was determined as livestock units per hectare and year (LU ha^-1^yr^−1^).

### Statistical analysis of activity patterns

The activity data consisted of position counts y_i_ of grazing, resting, and walking observations in each grid cell *i*. Because the data was over-dispersed, i.e. the variance in the data exceeded the mean, we assumed it to follow a negative binomial (NB) likelihood, which contains an additional free parameter κ to account for the degree of over-dispersion.

In addition, too many zeroes were present in the data as compared to the negative binomial distribution. Data with excessive zeros, so-called zero inflation (ZI), can be modeled by either a mixture model or a hurdle model ([33]: 11.3). A mixture NB model differentiates between a surplus of false zeroes and a NB distribution containing the true zeroes. A hurdle model separates zeroes and counts over zero in the first step and represents counts larger than zero by a NB distribution in the second step. Since we were interested in differences within the spatial intensity patterns rather than in presence/absence of the animals, we used a hurdle model. Hence, y_i_ ~ ZINB(μ_i_, π, κ), where parameter π determines the proportion of ZI as

$$ \Pr \left({\mathrm{y}}_{\mathrm{i}}\right)=\uppi \times {1}_{\left[\mathrm{y}=0\right]}+\left(1-\uppi \right)\times \mathrm{N}\mathrm{B}\left({\upmu}_{\mathrm{i}},\upkappa \Big|\mathrm{y}>0\right), $$

where κ is the over-dispersion parameter. If κ is large, the likelihood approaches a zero-inflated Poisson ([33]: 8.4).

In order to correct the intensity calculations for data gaps, we weighted each y_i_ by a weight w_i_ = ḡ/ḡ_i_, where ḡ = Σg_t_ Y^−1^ with g_t_ being the number of active GPS loggers at time t, and Y the total number of positions per study area. The denominator ḡ_i_ = Σg_ti_ y_i_^−1^ where g_ti_ is g_t_ assigned to each position x_it_. Cells with a greater than average number of loggers have w_i_ < 1, cells with fewer than average loggers have w_i_ > 1 and the average of all w_i_ is 1.

The third challenge with the data is their spatial dependence [27, 34]. It is likely that the intensity in a given cell is more similar to the intensity in neighbouring cells than in cells farther away. This spatial autocorrelation was included in the deterministic part of the model, which was log(μ_i_) = β**X** + f(s_i_) + f(z_i_) + ε_i_, where **X** was a matrix of centered and standardized covariates with associated coefficients β, f(s_i_) was a spatially structured error, f(z_i_) a non-linear effect of elevation, and ε_i_ a spatially unstructured error [26]. A two-dimensional random walk of second order was used as model for f(s_i_) ([35]: 3.4.2) and a random walk of first order was used for f(z_i_) ([35]: 3.3.1). Distances to shed and to water sources were log-transformed prior to analysis.

Since the estimation of such complex models is highly challenging using maximum likelihood, we used the recently developed INLA approach [30]. This method allows for fast and accurate inference for complex models and offers much flexibility with regard to the available likelihood of the data, as well as to random factors accounting for error covariance, e.g. spatial autocorrelation [26]. Since INLA operates in a Bayesian framework, all parameters require prior distributions, which in this case, were specified to be diffuse. The random effects f(s_i_), f(z_i_), and ε_i_ each depend on single precision parameters τ_s_, τ_z,_ and τ_ε_, respectively, which determine the smoothness of the effects [26, 36]. The choice of priors for the precision parameters is delicate because an arbitrarily flexible spatial effect could mask any effects of covariates. Therefore we scaled the reference standard deviations of τ_s_ and τ_z_ to 1 in order to achieve the same degree of smoothness for all effects [36]. We then evaluated a range of possible parameter combinations for the Gamma priors to τ_s_ and τ_z_ (Table 2) to assess the sensitivity of the posterior estimates to the prior choice [37]. The prior for τ_ε_ was chosen to be a Gamma distribution with shape 0.5 and rate 0.00149 in line with earlier studies [36–38]. All calculations were done in R 3.1.1 [39] using the r-inla package. A data subset and an example code are available as additional files (see Additional files 2 and 3).

**Table 2:** Evaluated priors for the scaled precisions of f(s_i_) and f(z_i_)

a	b	U
1	1.00E-05	0.1
**1**	**0.00025**	**0.5**
1	0.001	1
1	0.025	5
5	0.185	0.5
12	1.01	0.5
20	2.24	0.5

The values are the shape a and the inverse-scale b of the gamma priors for τ_s_ and τ_z_ (the precisions of the spatially structure effect f(s_i_) and the non-linear effect of elevation f(z_i_)), as well as the upper limit of the marginal standard deviation of the prior models U. For scaled models, a and b define U [36]. The prior value used for the rest of the results is shown in bold

Separate regressions were calculated for areas and activities as well as for subsets of data collected from individual animals, during certain times of the day or during certain periods of the season. For this purpose, the dataset was separated into subsets associated with particular individuals, time periods of four hours each day and periods of 14 days during the season. In order to avoid unstable results due to an insufficient number of observations, subsets of data containing less than 10 % of all observations per area for individuals and less than 5 % of all observations for day time and season were not analyzed. Regressions for daytime and season were weighted as described above.

### Correlations between covariate effects and characteristics of study areas

Specific characteristics of the six study areas (Table 1) were correlated to the estimated mean covariate effects on the grazing, resting, and walking patterns, obtained from the spatial regressions. Characteristics calculated for each area included the median and standard deviation (SD) of slope and stocking rate, as well as the average stocking period per paddock and the percentage areal shares of nutrient-rich vegetation and sparse forage. Spearman’s rank correlation coefficients were used to indicate the strength of association.

### Normalization of activity patterns

In order to compare the activity patterns derived from the GPS measurements between study areas and across activities, as well as with the average stocking rate per paddock, these data had to be normalized. This was achieved by transforming GPS position counts *y*_*i*_ in cell i into intensities R_i_ in LU ha^−1^∙yr^−1^ as:

$$ {R}_i=\left(N\times {n}_i\times P\right)\times {A_i}^{-1}, $$

where the portion of cows in cell i is *n*_*i*_ *= (y*_*i*_*∙ Y*^*−1*^*) ∙ w*_*i*_ with *Y* being the total number of positions, and *w*_*i*_ being the weight of the position counts, as described above. N is the size of the herd. *P* is the total grazing period on the study area and *A*_*i*_ = 0.0625 ha, the cell size of the grid.

## Results

### Characteristics of the position dataset

Per study area, between 120,000 and 485,000 GPS positions were recorded during time on pasture (Table 1). Because animals spent more time in the shed in study areas A-C in the Obwalden region, less positions were retained than in study areas D-F in the Lower Engadine region. In addition, the number of grazing days (due to specific climatic and pasture conditions in the study areas) and the number of cows equipped with a GPS collar differed between areas.

**Fig. 2:**
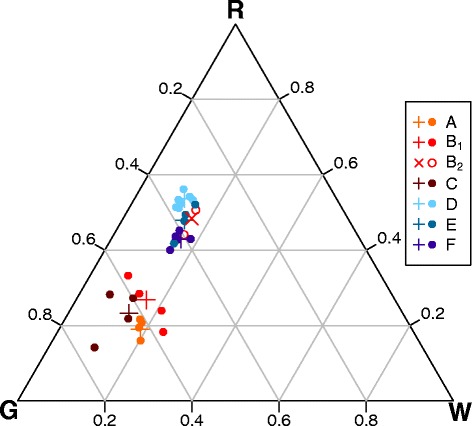
Percentage abundance of animal activities on pasture. Abundances of the activities grazing (G), resting (R) and walking (W) at pasture are shown for individual tracked dairy cows (circles) and all observations (crosses) in the six areas A-F. Time at pasture for dairy cows was limited to daytime in areas A-C. Suckler cows in area B, which spent the night outside are marked separately by open circles (B_2_)

Differences in the relative abundance of the activities of grazing, resting, and walking were mainly associated to night sheltering in the two regions (Fig. 2). Dairy cows in the Obwalden region (A-C), which spent the night in the shed, most frequently grazed (55 % - 75 % of the positions), followed by resting (14 % - 33 %), and walking (7 % - 24 %). In area B, suckler cows (B_2_) were clearly different from dairy cows (B_1_), because they did not spend time in the shed. In the region of the Lower Engadine (areas D-F), where cows stayed outside during the night, resting positions were most frequent (40 % - 56 %), followed by grazing (33 % - 45 %), and walking (10 % - 18 %). Differences in the activity budgets between individuals were generally smaller than between study areas.

### Average stocking rate and fine-scale activity patterns

**Fig. 3:**
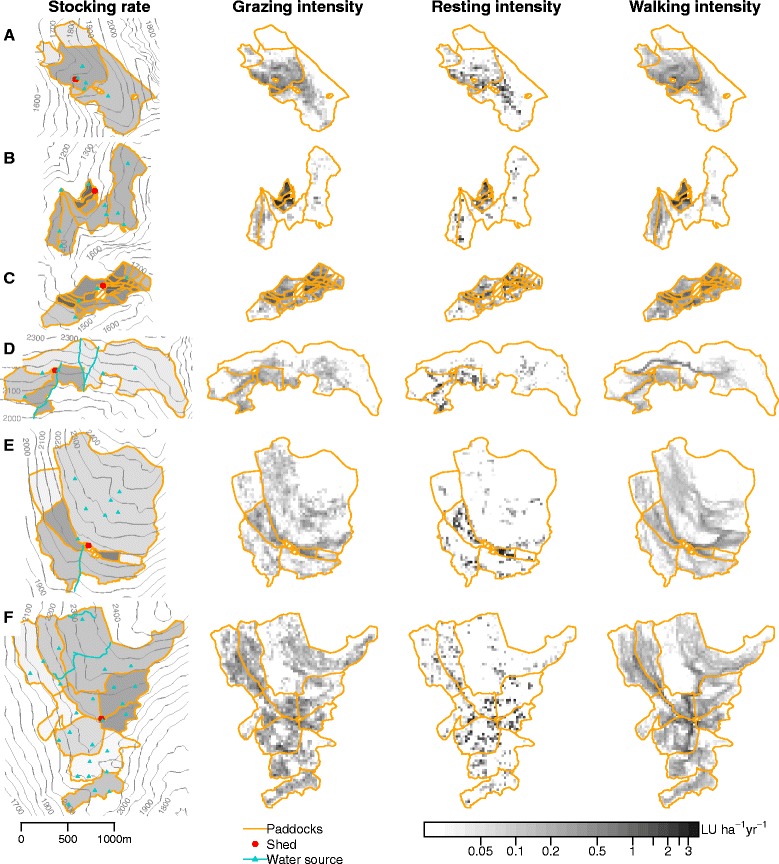
Stocking rate and fine-scale activity patterns in six study areas A-F. Stocking rates at paddock scale were obtained from interviews with herdsmen. Fine-scale intensities of grazing, resting, and walking were obtained by discretizing GPS positions on a 25 m grid and normalizing to LU ha^−1^ yr^−1^ using herd size and the duration of summering. Paddock borders, sheds and water sources are also displayed. Small, hatched paddocks are areas excluded from grazing. Contour lines are based on elevation data of swisstopo, Wabern, Switzerland. All areas are drawn to the same scale

There were striking differences between the average stocking rate per paddock and the GPS-based fine-scale patterns of grazing, resting, and walking intensity (Fig. 3). Within paddocks, there were large differences between areas intensively used by the cows and areas that were avoided. The discrepancy between the intensity measures was especially evident in study areas with large average stocking periods per paddock. Nevertheless, considerable within paddock variability was even recognizable in areas with many paddocks, for example in area C.

The intensity patterns of grazing and walking were very similar, except for the long travelling routes of cows to distant pastures visible in the walking patterns in the large study areas of the Lower Engadine. Resting behavior showed a more punctual distribution, with intensity peaks often lying slightly outside of the most intensively grazed grid cells.

### Common drivers of grazing, resting, and walking intensity

**Fig. 4:**
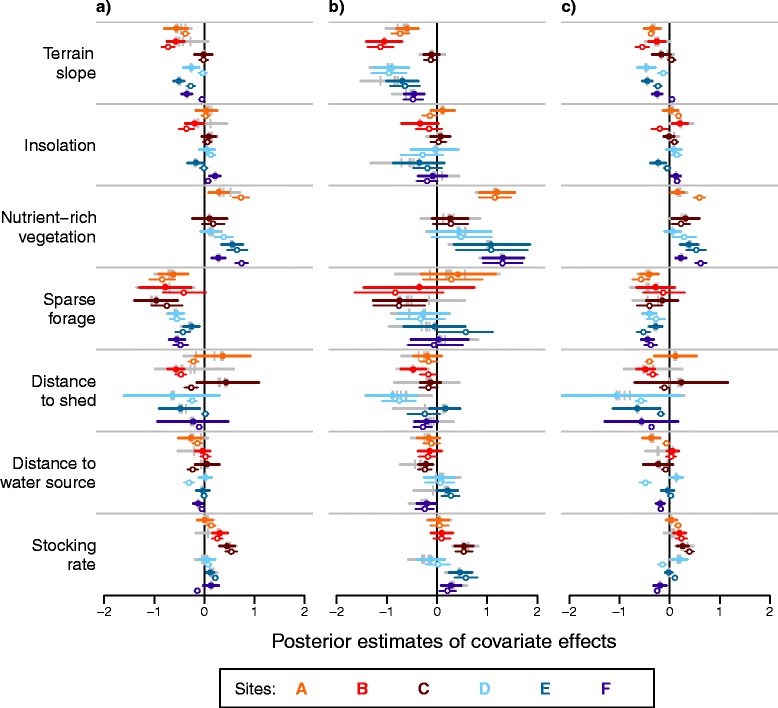
Estimated effects of six covariates on fine-scale activity patterns. The dots show the mean and the lines represent the 95 % credibility intervals of the estimated effects of standardized covariates on grazing (a), resting (b), and walking activity (c) in the six study areas A-F. Filled color symbols and bold lines are posterior estimates using a Gamma prior to τ_s_ and τ_z_ with shape 1 and rate 0.0025. Grey symbols represent the mean estimates using the other priors displayed in Table 2. The grey line is the joint range of the 95 % credibility intervals of all evaluated priors. Open symbols and thin colored lines show estimates and 95 % credibility intervals of the models without spatial terms, i.e. ignoring spatial autocorrelation

The estimated effects of environmental and management covariates on the intensity of grazing, resting, and walking agreed reasonably well across all six study areas (Fig. 4). The main determinants of grazing intensity were terrain slope, forage quality, and, with notable exceptions, stocking rate per paddock. Terrain slope had a significant negative effect on grazing in almost all areas (except area C). Grazing was more frequent on nutrient-rich vegetation than nutrient-poor vegetation, which was the baseline in all models (except for area B where the baseline of models was nutrient-rich vegetation). The positive effect of nutrient-rich compared to nutrient-poor vegetation was significant in three areas. Grazing was significantly less intense in patches of sparse forage in all areas. In three study areas, stocking rate per paddock (Fig. 3) had a significant positive effect on grazing, i.e. the grazing pressure imposed by the pasture rotation was observable in the distribution pattern.

Resting intensity was generally determined by the same covariates as grazing intensity with some notable differences. Significant negative effects of terrain slope and significant positive effects of nutrient-rich vegetation on resting were present in the same areas as for grazing. In contrast, a significant negative influence of sparse forage on resting was present in only one area, and stocking rate had a significant positive effect on resting in three areas.

Covariate effects on walking were relatively close to grazing. Terrain slope had a significant negative effect on walking in all areas. Nutrient-rich vegetation had a significant positive effect on walking in the same four areas as for resting. Sparse forage had a significant negative influence on walking in four areas, and stocking rate per paddock had a significant positive effect on walking in three areas, but a significant negative effect in area F. In this large study area, some paddocks are rarely grazed but frequently walked through.

The variables insolation, distance to shed, and distance to water sources mainly showed insignificant or diverging effects on the activity patterns in different study areas.

### Differences between study areas

Apart from the general effects unifying most of the study areas, specific covariate effects were only present in particular areas (Fig. 4). Most remarkably, there was no effect of terrain slope on grazing and resting intensity in area C. Area C was also the only study area that exhibited a significant negative effect of sparse forage on resting and one of the two study areas with no effect of sparse forage on walking. Nutrient-rich vegetation had no effect on any activity pattern in area D and no effect on grazing in area C. Stocking rate had no significant effect on either of the three activity patterns in area A. No significant effect of stocking rate on resting was present in area B or on grazing and resting in area D.

### Sensitivity of model results to spatial autocorrelation and prior choice

Estimates of fixed covariate effects may depend on the specification of the random error terms and, if fitted in a Bayesian context as done here using INLA, their associated prior distributions [26, 27, 37].

Specifying error terms to account for spatial autocorrelation had a large impact on the estimated covariate effects (open and closed symbols in Fig. 4). The significance and/or direction of several of the estimated effects changed depending on whether spatial autocorrelation was accounted for or not. Differences in distance effects were especially striking. If spatial autocorrelation was ignored, many effects of the distance to shed or water sources were estimated to be highly significant. In contrast, effects of terrain slope on grazing and walking were generally less prominent if estimated without spatial error terms.

In order to evaluate the sensitivity to prior choice, we tested a range of choices for the Gamma prior distribution of the precision parameters for the spatially structured random effect and the non-linear effect of elevation (Table 2). The analysis showed that in most cases the direction and strength of the fixed effects were robust to different prior choices (grey marks in Fig. 4). Fixed effects were slightly sensitive to the choice of prior parameters in areas A and B and in the models of resting intensity, only. In these cases, the data contained insufficient information to constrain the random effects away from their prior distributions, thereby resulting in different estimates of fixed covariate effects.

### Estimates of additional model parameters

Besides the fixed covariates effects, five other parameters were estimated for the regression models of each activity and study area (Table 3). Small values of NB parameter κ indicated over-dispersion for resting, especially in areas D-F, where patterns were clumped. In other cases, with high values of κ, a Poisson model could be used. Part of this clumped pattern was also captured by the proportion of ZI π, which amounted to over 60 % of zeros in the position data for resting, mostly around 30 % for grazing and between 0 and 36 % for walking.

**Table 3:** Estimated parameters in regression models fitted per area and activity

Activity	Parameter	A	B	C	D	E	F
G	κ	2.83 (0.26)	25.9 (4.64)	18.3 (3.75)	2.3 (0.18)	1.61 (0.0802)	2.02 (0.0989)
G	π	0.306 (0.016)	0.366 (0.018)	0.064 (0.012)	0.337 (0.0123)	0.298 (0.0113)	0.208 (0.0081)
G	τ_s_	0.075 (0.021)	3780 (3700)	0.325 (0.212)	0.0302 (0.008)	0.25 (0.0763)	0.0235 (0.0036)
G	τ_z_	3800 (3710)	3590 (3600)	3650 (3670)	11.7 (15.6)	4770 (5200)	3780 (3710)
G	τ_ε_	539 (542)	0.605 (0.053)	2.21 (0.307)	421 (485)	708 (748)	640 (645)
R	κ	0.469 (0.042)	19 (4)	0.764 (0.065)	0.0032 (0.002)	0.003 (0.0017)	0.0023 (0.001)
R	π	0.474 (0.017)	0.592 (0.019)	0.184 (0.018)	0.554 (0.013)	0.683 (0.0107)	0.576 (0.0098)
R	τ_s_	3720 (3670)	3750 (3680)	3540 (3580)	3890 (3790)	3620 (3630)	3690 (3670)
R	τ_z_	3710 (3680)	3810 (3710)	3730 (3680)	3430 (3590)	3790 (3720)	3540 (3620)
R	τ_ε_	131 (127)	0.34 (0.0373)	122 (242)	296 (382)	346 (427)	370 (439)
W	κ	11.9 (1.75)	23.4 (4.34)	18.8 (3.53)	7.43 (1.3)	3.62 (0.253)	6.26 (0.562)
W	π	0.269 (0.015)	0.369 (0.018)	0.038 (0.009)	0.3 (0.0119)	0.268 (0.0109)	0.178 (0.0077)
W	τ_s_	0.088 (0.017)	3850 (3730)	0.043 (0.006)	0.0105 (0.002)	0.0812 (0.015)	0.014 (0.0014)
W	τ_z_	2.76 (1.28)	4.24 (3.11)	3810 (3730)	1.02 (0.291)	4.59 (2.09)	1.38 (0.355)
W	τ_ε_	767 (641)	1.22 (0.126)	383 (386)	500 (523)	894 (669)	784 (643)

Values are estimates for the over-dispersion parameter κ, the proportion of zero inflation π, the precision of the spatially structured effect τ_s_, the precision of the non-linear effect of elevation τ_z_, and the precision of the spatially unstructured random error τ_ε_ for the activities grazing (G), resting (R), and walking (W) in six study areas A-F

Spatial autocorrelation was mostly accounted for by the spatially structured effect, as indicated by low values of the precision parameter τ_s_ and thus high variance. A notable exception was area B, where most of the spatial structure was either captured by a non-linear trend of elevation (low precision τ_z_) or the spatially unstructured random error (low precision τ_ε_).

### Covariate effects estimated for individual animals, daytime and season

**Fig. 5:**
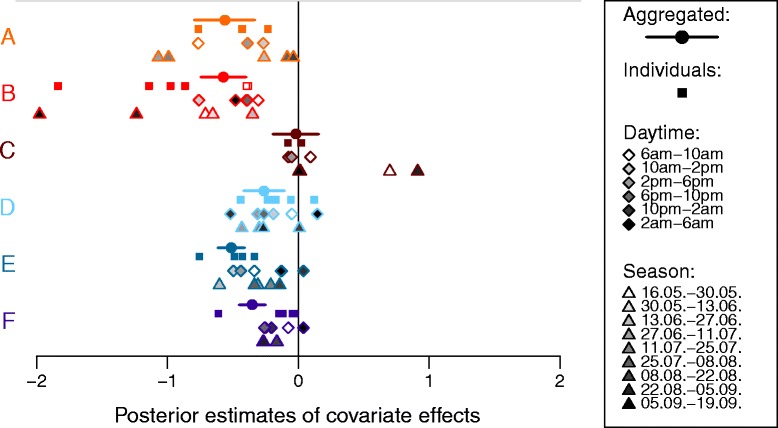
Effects of terrain slope on grazing as affected by individuals, daytime and season. Symbols show estimated effects of terrain slope on grazing intensity in the six study areas A-F (colors identical to Fig. 4) for the entire dataset (as in Fig. 4) and different subsets of the data: squares are estimates for individual animals, open squares in area B show suckler cows, rhombi are estimates for four hours periods with increasing gray shade from 6 am and circles are estimates for 14 days periods. All estimates were obtained using Gamma priors to τ_s_ and τ_z_ with shape 1 and rate 0.0025

Covariate effects estimated for subsets of the data generally agreed with the results obtained for the aggregated data per area (Fig. 5 and Additional file 4 with effects of all covariates and activities). Individual variation in the response of grazing intensity to terrain slope (squares in Fig. 5) was relatively small in most areas and not too far from the uncertainty range estimated from the aggregated data. An exception was area B, where dairy and suckler cows were tracked and grazing by suckler cows responded less to terrain slope than dairy cows. Variation of estimated slope effects with daytime was in the range of individuals. No consistent patterns across areas were recognizable, with the exception of some trend towards weaker slope effects at late night (black rhombi) in areas D-F, where cows grazed outside at night. The seasonal variation of slope effects was strongly dependent on the particular area, and again no consistent pattern was observable across areas. Seasonal differences between areas may at least partly be due to the pasture rotation, specific to each area. Again, seasonal variation was strongest in area B due to differences between dairy and suckler cows.

Across all covariates and activities, a large variation in estimated effects between individuals, daytime or season coincided with a larger uncertainty of the effects estimated for the aggregated data (Additional file 4). For example, this was evident for effects of sparse forage, which are highly uncertain for resting using the aggregated data and show a high variation between individuals, daytime and season using the subsets of data.

### Correlations of covariate effects with characteristics of study areas

**Fig. 6:**
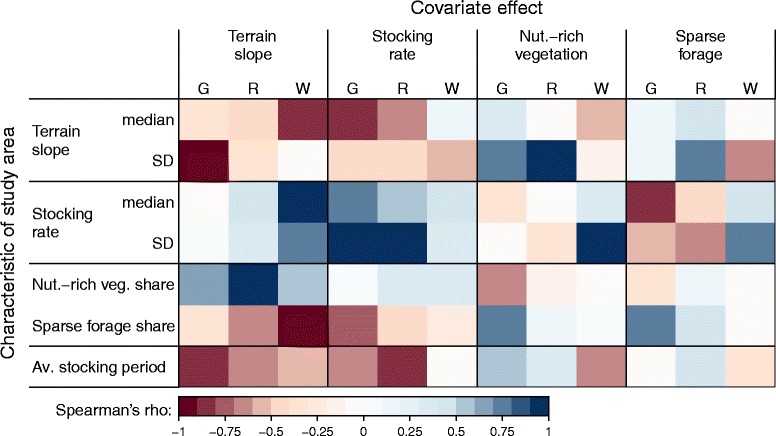
Strength and direction of association between covariate effects and characteristics of study areas. Colours represent Spearman’s rank correlation coefficients between covariate effects (the estimated regression coefficients for the activities of grazing (G), resting (R), and walking (W) displayed in Fig. 4) and characteristics of the study areas (medians and standard deviations of slope and stocking density and the average stocking period per paddock given in Table 1, as well as the areal share of nutrient-rich vegetation and sparse forage)

We tested various characteristics of the study areas for their ability to explain the rank-order of covariate effects across areas (Fig. 6). Only those covariates that were significant in the majority of models, namely terrain slope, stocking rate, nutrient-rich vegetation and sparse forage, were evaluated.

Weak slope effects on resting and walking intensity were associated with high median and SD of stocking rate; strong negative slope effects on resting and walking were present in areas with a high areal share of sparse forage. A stronger negative slope effect on grazing and resting was found where the average stocking period was long. Large positive effects of stocking rate were present in areas with a high median and SD of stocking rate. The effect of stocking rate on grazing and resting was weaker in areas with a high share of sparse forage and long average stocking periods.

Positive effects of nutrient-rich vegetation were related to a large terrain slope SD in the case of grazing and resting, and to a high SD of stocking rate in the case of walking. Grazing was more affected by nutrient-rich vegetation in areas where the average stocking period was long. Negative effects of sparse forage on grazing intensity were associated with a high median stocking rate. The negative effect of sparse forage on grazing was weaker where the areal share of sparse forage was high.

## Discussion

The analysis of fine-scale spatial activity patterns of grazing livestock on heterogeneous subalpine pastures revealed common and area-specific drivers, from which implications for livestock management can be derived. The data also demonstrate the importance of spatial autocorrelation in the analysis of activity patterns.

### Challenges in the analysis of livestock activity patterns

Quantification of animal activity patterns is greatly facilitated by bio-logging systems, such as the employed GPS tracking. This yielded fairly accurate (absolute accuracy of around ±3 m) position records over extended periods of time at a temporal resolution which allowed behavioral classification [19]. While recording positions was relatively straightforward, statistical analysis of position data posed a number of challenges, for instance the existence of spatial autocorrelation and of data gaps, in addition to issues arising from the employed statistical technique, such as over-dispersion, ZI, or effects of prior choice.

The study benefited of the recently developed INLA method [30], which provides fast and accurate inference for a large range of fairly complex models. It can accommodate many of the common difficulties with the analysis of ecological data, namely autocorrelation structures or count data [26, 27]. In INLA, spatial autocorrelation is included in a hierarchical way as a spatially structured random effect, which describes the dependence among neighboring cells not explained by the covariates [27]. There is a range of options to define the spatial random effect depending on whether the neighborhood is represented as a regular grid or an irregular lattice. On a regular grid, the spatial field can be represented by a second-order random-walk model in two dimensions, i.e. the value in a particular cell depends on the values of the four cells cardinally adjacent, diagonally adjacent, and cardinally adjacent of second order ([35]: 3.4.2). This approximates a thin-plate spline [40] and flexibly captures trends in two dimensions not described by covariates. In addition, we have included a smooth non-linear representation of elevation, resulting in a directed trend along the elevational gradient. The estimated parameter values show that both trends are, to a certain degree, exchangeable, depending on the particular topographic setting of the study areas. For example, grazing activity is spatially autocorrelated in five areas and determined by elevation in area B. There, the farm shed is located near the bottom of the grazing area and hence, elevation relative to the shed explains a substantial part of the spatial pattern.

The comparison of parameter estimates with and without spatial autocorrelation corroborates earlier evidence of its importance [27, 34]. Many estimated effects were biased in significance, which was either inflated or too conservative, and several even in direction. Especially striking are differences in the significance of effects by distances to shed and water, which are strongly spatially autocorrelated and therefore largely accounted for by the spatial random effect. Ignoring spatial autocorrelation in spatially discretized data inflates the significance of effects by (arbitrarily) increasing the number of samples and treating them as independent observations [34].

Our results also show that over-dispersion and zero-inflation need to be considered if analyzing position data from heterogeneous landscapes. Models may be seriously over-dispersed, especially where observations are clumped (as for resting), and the data may contain up to 60 % of zeros. As previously discussed, one of the greatest advantages of INLA is the ease of adjusting the likelihood to the properties of the response variable in order to account for over-dispersion and zero-inflation, if necessary.

Since INLA is a Bayesian method, priors need to be specified. While this may be an advantage if information content in the data is low and parameter ranges are well-known in advance [41], it maybe of considerable concern if little is known beforehand [38]. Special care has to be paid to the precision priors of the random effects [26, 27, 37]. In the present study, we found little influence of prior choice on the estimates of fixed covariate effects, in which we were most interested in. As expected, larger effects were found where parameters were less defined by data, for example in area B, and for additional parameters of the model, for example the precisions of the spatial and elevational trends.

Finally, we had to deal with data gaps, which are common to ecological field studies. The employed regression model allowed for weighing of the observations entering the likelihood calculation. Observations during periods with device failures were given a higher weight because they were representative of a larger number of animals. Nevertheless, model evaluations showed that the weighing procedure was of minor importance for the estimated effects.

### Common influences of the environment on livestock activity patterns

Understanding and controlling livestock distribution is of major importance in heterogeneous and rugged landscapes. Because natural patterns of increased resource availability are likely to be reinforced by the animals’ utilization patterns [10, 42], inappropriate pasture management may result in weed expansion [5, 43] or shrub encroachment [14]. Terrain slope and vegetation type were found to be main environmental determinants of cattle distribution within a number of previous investigations [42, 44–46]. These former studies often concentrated on assessing one covariate, they were mostly conducted at one or two study sites, and analyzed grazing distribution exclusively [23, 42, 44] or general pasture utilization without discriminating between activities [45]. The present study, comprising six study areas and differentiating between patterns of grazing, resting, and walking, generally corroborated these earlier findings: in all six study areas, grazing was significantly reduced in patches of sparse forage, and in five areas, grazing was reduced at steeper terrain slopes, reflecting optimal foraging behavior for spatially distributed food resources. Common to all areas was the negative effect of terrain slope on walking, and in five areas on resting activity. By modelling the spatial dependence within the activity patterns and by using an additive model, we were able to extract the separate effect of each covariate. Although slope gradient is often negatively correlated with forage quality, we found that each of the two covariates had an own effect on livestock distribution.

Different activities of livestock have distinct impacts on the pasture ecosystem [2], suggesting that patterns of each activity should be specifically analyzed. Kohler et al. [29] reported distinct spatial distributions of three forms of cattle impact on the pasture, namely herbage removal, dung deposition, and trampling, which are predominately associated to grazing, resting, and walking in the present study, respectively. We found less pronounced differences between the patterns of these activities, likely because estimation of impacts in the field confounds livestock activity with the sensitivity of different pasture areas to this activity. The most noticeable differentiation we found was the plane distribution of grazing, the very punctiform distribution of resting, and the linearly structured distribution of walking. Grazing and walking were spatially correlated to some degree, because animals walk short distances between sequences of forage intake. Since walking is a relatively rare activity and its distinction from grazing is challenging based on GPS positions alone [19], future studies of pasture use may consider differentiating grazing and resting only.

Differences between activity patterns also depended on the study area. Distinct core areas of walking were evident in the extended study areas in the Lower Engadine region, where cows had a long walk to the most distant pastures. In general, patterns of the three activities were related to a somewhat different set of covariates. For example, in contrast to grazing, resting was not affected by the presence of sparse forage, with the exception of area C, where the patches with sparse forage were mainly wetlands.

Differences between areas were stronger than between individual animals, which generally varied little with regard to activity budgets or estimated covariate effects. The large difference in activity budgets and covariate effects between dairy and suckler cows in area B supports the finding that similarly managed animals show similar response to environmental conditions. The fact that studied animals needed to be well-integrated into the herd, may have favored homogeneous results. Still, some differences are visible and would merit more detailed investigations after selecting animals with contrasting characteristics [47]. Estimated covariate effects for subsets of particular daytime and season periods indicate some variation but no consistent patterns across areas. Differences between areas can mostly be explained by night sheltering. This corroborates the generality of the effects estimated using the aggregated data per area.

### Implications for pasture management

The fact that several covariate effects were not consistent over all six study areas allows deriving some practical recommendations for the management of upland pasture areas. The results show that the magnitude of covariate effects on activity patterns depended on the specific context of the study area. For example, the availability of nutrient-rich vegetation favored the avoidance of vegetation with sparse forage supply or a large variability of terrain slopes increased the magnitude of the slope effect.

Study areas were preselected along a gradient of livestock management as expressed by the placement of water ponds and the rotation of paddocks. The distance to water only had a minor effect on overall livestock activity in most areas. Interestingly, the effect was significantly negative in area F, which had a relatively high density of water sources. This suggests that distance to water was not limiting livestock activity in any of the study areas, which is in contrast to findings from other studies operating at larger scales [24, 25]. Still, the effect in area F demonstrates that watering places may serve as a tool for grazing management if available in a sufficient density [21].

The second management measure, the average stocking period per paddock was associated with a reduced negative effect of terrain slope on grazing activity. Hence, a frequent grazing rotation with short stocking periods per paddock is recommended to counteract the inhibiting effect of terrain slope. Interestingly, there was no association of the average stocking period with the negative effect of sparse forage, suggesting that not all environmental limitation can be simply overcome by appropriate pasture management in these highly heterogeneous landscapes.

## Conclusions

Our investigation highlights that bio-logging data need to be analyzed using appropriate statistical techniques. INLA provides fast inference for complex models and allows analyzing prior sensitivity and data subsets by reducing computation time from hours to minutes as compared to traditional Markov Chain Monte Carlo. Our calculations showed that ignoring spatial autocorrelation in our regression analyses strongly altered estimated covariate effects. If we had not considered spatial autocorrelation, we would have reached different conclusions, likely with a major emphasis on effects of distance to shed and water. These two variables are strongly spatially autocorrelated and therefore sensitive to the violation of statistical assumptions. Hence, earlier conclusions of GPS studies analyzed without the consideration of spatial autocorrelation should be interpreted with care. In view of the ecological interpretation of the results, we found that livestock activities on subalpine pastures were primarily controlled by environmental conditions, mainly terrain slope and vegetation. Although the activities grazing, resting and walking were generally influenced by similar factors, several differences suggest investigating activity-specific impacts of livestock on the ecosystem. Our results also demonstrate that a frequent pasture rotation can alleviate the inhibiting effects of the environment to some degree, leading to better resource use in topographically unfavorable pasture areas.

### Availability of supporting data

The data set supporting the results of this article is included as additional file 2.

## Additional files

Additional file 1:
**Contains the mapped vegetation types and their aggregation into 3 groups.** (PDF 6 kb) Additional file 2:
**Contains the dataset of study area A.** (TXT 184 kb) Additional file 3:
**Contains example R code for the spatial regression with INLA.** (TXT 2 kb) Additional file 4:
**Contains a figure displaying all covariate effects on animal activities as affected by individuals, daytime and season.** (PDF 80 kb)

## Abbreviations

GPS: Global Positioning System
ha: hectare = 10000 m^2^
INLA: Integrated Nested Laplace Approximation
LU: Livestock Unit
NB: Negative Binomial
No: Number
SD: Standard Deviation
ZI: Zero Inflation
