# Supplementary material for: Patterns of livestock activity on heterogeneous subalpine pastures reveal distinct responses to spatial autocorrelation, environment and management
Source: Mov Ecol. 2015 Oct 8;3:35. doi: 10.1186/s40462-015-0053-6 (PMC4598957; doi:10.1186/s40462-015-0053-6)
Supplement: Additional file 1: — Contains the mapped vegetation types and their aggregation into 3 groups. (PDF 6 kb) [file 40462_2015_53_MOESM1_ESM.pdf]

Additional file 1: Mapped vegetation types at alliance level and aggregation into 3 groups.

Type	English name	Latin name	Aggregated group
1	Blue moorgrass-evergreen sedge slopes	Seslerion	Nutrient Poor
2	Northern rusty sedge grasslands	Caricion ferruginae	Nutrient Poor
3	Mat-grass swards and related communities	Nardion	Nutrient Poor
4	Subalpine Small Reed meadows	Calamagrostion	Nutrient Poor
5	Atlantic and Sub-Atlantic humid meadows	Calthion	Nutrient Rich
6	Mountain and subalpine hay-meadows	Polygono-Trisetion	Nutrient Rich
7	Mesophile pastures	Cynosurion	Nutrient Rich
8	Rough hawkbit pastures	Poion alpinae	Nutrient Rich
9	Hercynio-alpine tall herb communities	Adenostylion	Nutrient Rich
10	Alpine dock communities	Rumicion alpini	Nutrient Rich
11	Large sedge beds	Magnocaricion	Sparse forage
12	Acidic fens	Caricion fuscae	Sparse forage
13	Rich fens	Caricion davallianae	Sparse forage
14	Crooked-sedge and related communities	Caricion curvulae	Sparse forage
15	Shrubby clearings	Sambuco-Salicion	Sparse forage
16	Willow brush	Salicenion waldsteinianae	Sparse forage
17	Juniperus nana scrub	Juniperion nanae	Sparse forage
18	Alpenrose heaths	Rhododendro-Vaccinion	Sparse forage
19	Dwarf Azalea and Vaccinium heaths	Loiseleurio-Vaccinion	Sparse forage
20	Eastern siliceous larch and Arolla forests	Larici-Pinetum cembrae	Sparse forage
21	Fine calcareous screes	Petasition paradoxi	Sparse forage
22	Not Accessible	-	Sparse forage
