# Supplementary material for: Patterns of livestock activity on heterogeneous subalpine pastures reveal distinct responses to spatial autocorrelation, environment and management
Source: Mov Ecol. 2015 Oct 8;3:35. doi: 10.1186/s40462-015-0053-6 (PMC4598957; doi:10.1186/s40462-015-0053-6)
Supplement: Additional file 4: — Contains a figure displaying all covariate effects on animal activities as affected by individuals, daytime and season. (PDF 80 kb) [file 40462_2015_53_MOESM4_ESM.pdf]

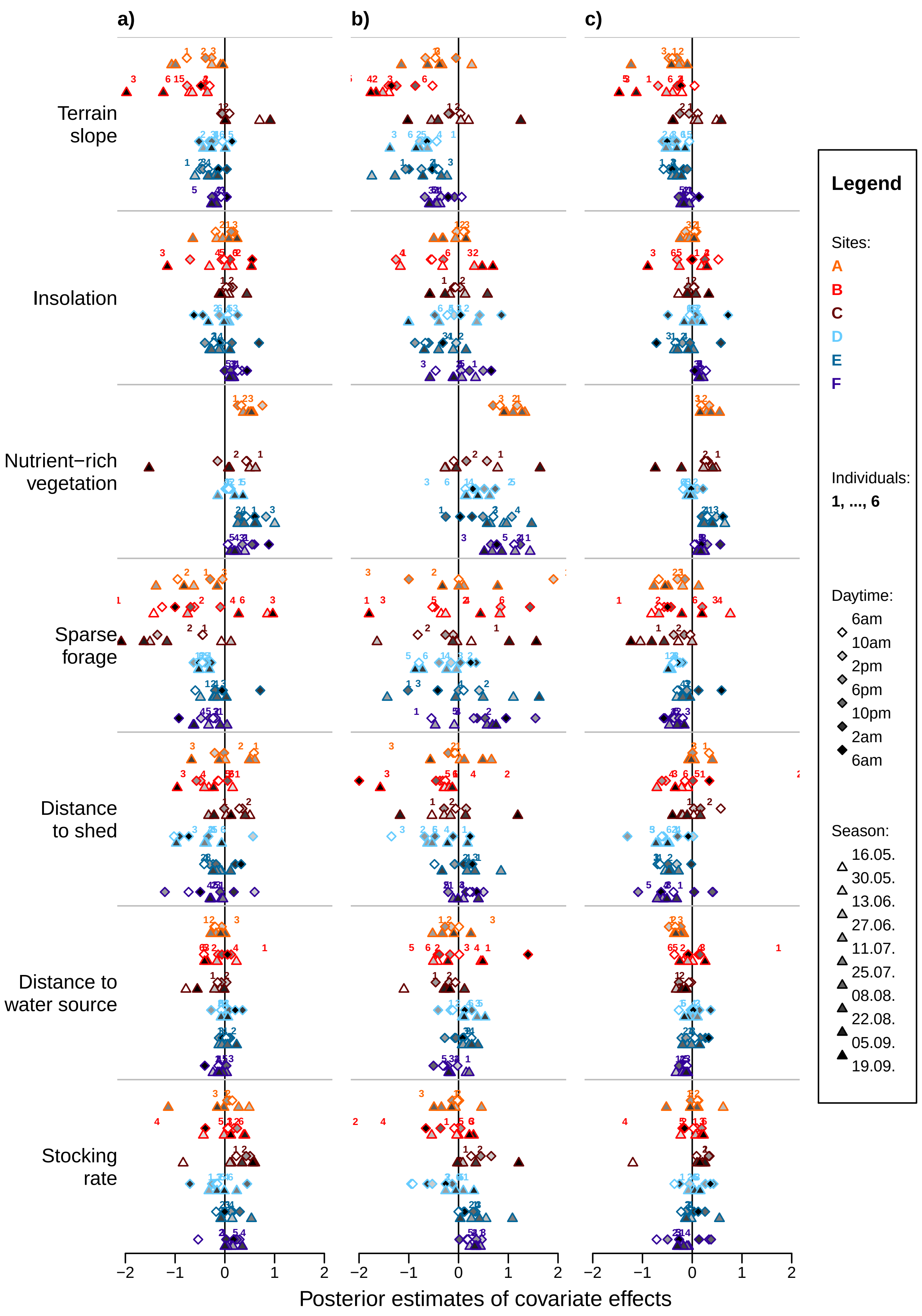

Additional file 4. Estimated covariate effects as affected by individual animals, time periods during the day and during the season. Symbols show mean estimated covariate effects on grazing (a), resting (b), and walking activity (c) in the six study areas A-F (colors identical to Figure 4) for different subsets of the data: squares with numbers are estimates for individual animals, rhombi are estimates for 4 hours periods with increasing grayshade from 6am and circles are estimates for 14 days periods. All estimates were obtained using a Gamma prior to τ_s and τ_z with shape 1 and rate 0.0025.
